# Supplementary material for: Complex‐centric proteome profiling by SEC‐SWATH‐MS
Source: Mol Syst Biol. 2019 Jan 14;15(1):e8438. doi: 10.15252/msb.20188438 (PMC6346213; doi:10.15252/msb.20188438)
Supplement: Supplementary file 8 — Dataset EV7 [file MSB-15-e8438-s008.zip › feature_plots_string/P21399.pdf]

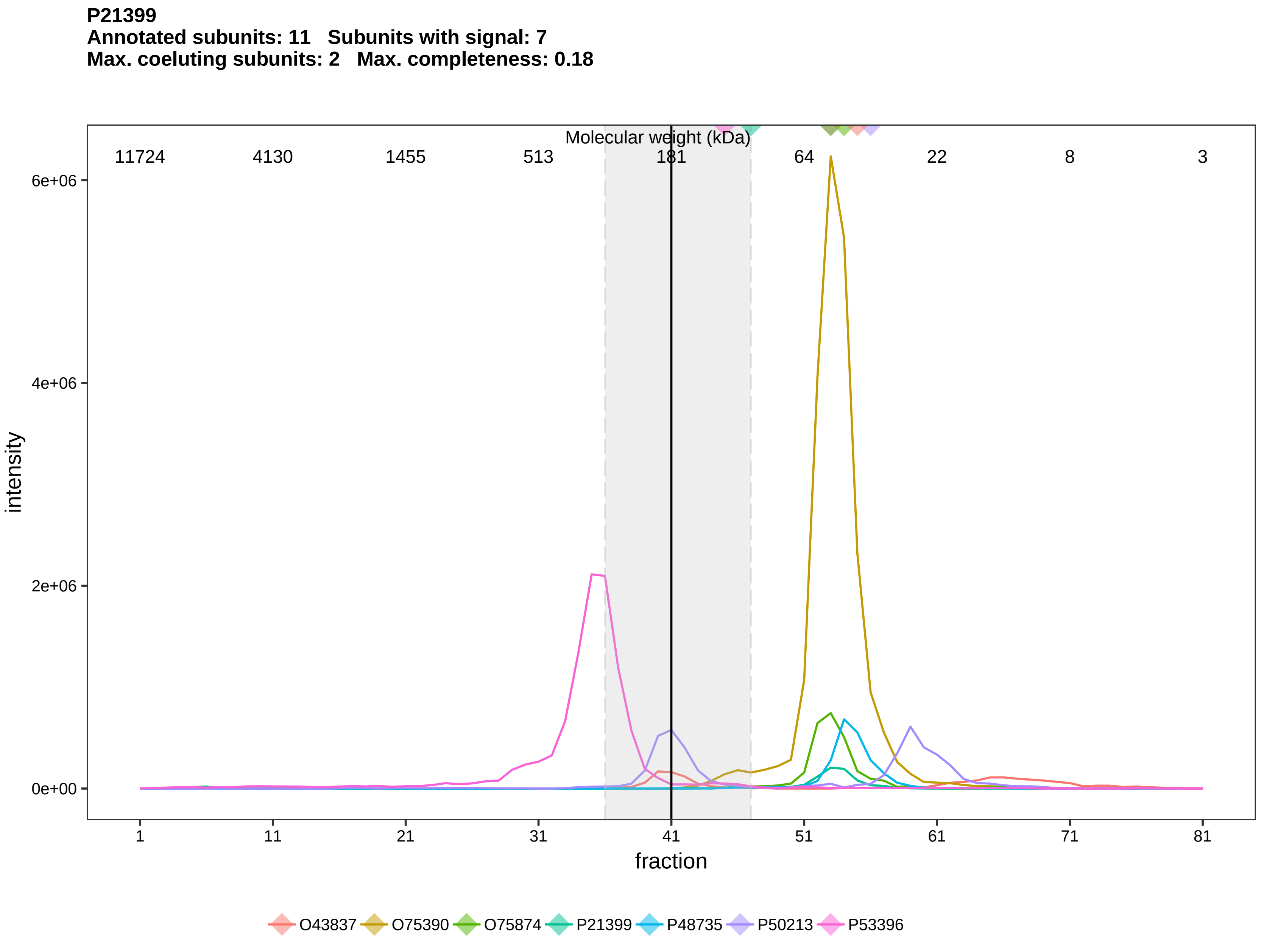

P21399
Annotated subunits: 11 Subunits with signal: 7
Max. coeluting subunits: 2 Max. completeness: 0.18
Molecular weight (kDa)
11724
4130
1455
513
181
64
22
8
3
6e+06
4e+06
2e+06
0e+00
intensity
1
11
21
31
41
51
61
71
81
fraction
O43837
O75390
O75874
P21399
P48735
P50213
P53396
